# Drug classification for patients with comorbidities

**DOI:** 10.1186/s40545-022-00453-9

**Published:** 2022-09-22

**Authors:** Farid Belialov

**Affiliations:** Department of Gerontology, Geriatrics, and Clinical Pharmacology, Russian Medical Academy of Continuous Professional Education, Yubileiny 100/4, Irkutsk, 664079 Russia

**Keywords:** Comorbidity, Multimorbidity, Drug classification, Coronary artery disease, Cirrhosis, Mental disorder

The development of new effective drugs along with population aging has led to a high number of patients with two or more chronic diseases. In the presence of multiple comorbidities, a drug selection is a difficult task for physicians. When choosing drugs, one should take into account the possible impact of treatment on comorbid diseases, which can be positive or negative (Table 1). In addition, polypharmacy can lead to various drug-induced disorders and drug–drug interactions. The long lists of contraindications, warnings, and precautions in drug instructions poses difficulties for physicians to select optimal drugs for the treatment of patients with comorbidities.

**Table 1:** Effect of drugs on comorbid diseases

Drugs	Diseases						
	CAD	Hypertension	AF	Diabetes	CKD	Heart failure	Cirrhosis
ACEI, ARB	+	+ +	+	+	+ +	+ +	
Aldosterone antagonists		+	+		+ /−	+ +	+ +
Beta-blockers	+ +	+ +	+ +			+ +	+ +
Ranolazine	+ +		+ +	+			− −
Statins	+ +		+	+ + /−	+		+
Metformin	+			+ +	+	+	
SGLT2	+	+		+ +	+ +	+ +	

*ACEI* angiotensin-converting-enzyme inhibitor, *AF* atrial fibrillation, *ARB* angiotensin receptor blockers, *CAD* coronary artery disease, *CKD* chronic kidney disease, *SGLT2* sodium–glucose cotransporter 2 inhibitors, + + favourable effect, + possible effect, − undesirable effect, − − unfavourable effect

Classification systems uniting items with similar features are useful for practical purposes. Drug classifications with the positive and negative labeling had been developed for specific patient populations or clinical settings. For instance, drug selection in pregnant women which is based on the FDA classification system and divides all drugs into classes A, B, C, D, and X depending on the risk to the fetus and newborn. The FDA not supported this classification from 2015, but its modifications are used until now in a few countries. FORTA list which combines positive and negative labeling (A, B, C, and D) of medications chronically prescribed to older patients had been proposed to reduce polypharmacy [1]. The Arizona Center for Education and Research on Therapeutics (AzCERT) maintains the drug list that has a risk of QT prolongation and cardiac arrhythmias. This classification divides drugs into groups with conditional, possible, known and high risk of malignant arrhythmias.

In this paper, it is suggested to rate the effects of drugs on comorbidities as favorable, possible, neutral, undesirable, and unfavorable (Table 2). In addition, these drug classes can be labelled as A, B, C, D, and X. Classification systems mentioned above can be integrated into the FORCOM (Fit fOR COMorbidity) universal drug classification system which will be include the effects of different drugs on comorbidities. Besides, due to the specific and multidirectional drug–disease interactions, it will be require descriptions and commentaries for practice use.

**Table 2:** FORCOM drug classification according to influence on comorbid diseases

Class	Drug’s effect	Influence on comorbid disease
A	Favourable	Drugs with pronounced positive effects
B	Possible	Drugs may have a mild positive effects
C	Neutral	Drugs without any significant effect or contradictory effect on comorbid disease
D	Undesirable	Drugs with possible rare non-severe adverse effects or unexplored drugs
X	Unfavourable	Drugs with high incidence (≥ 1%) of severe adverse effects

The paucity of randomized trials in patients with overt comorbidities makes it difficult to develop drug classifications. Patients with severe comorbidities are generally excluded from trials to allow more objective investigations of drugs and to reduce the number of adverse effects. Unfortunately, subgroup analyses of randomized clinical trials are not reliable. Information on drug classification is mainly based on observational studies and registries.

An example of the FORCOM classification coronary artery disease medication in patients with liver cirrhosis is presented in Table 3. Decompensation of cirrhosis is prevented by nonselective β-blockers (nadolol, propranolol, and carvedilol) which are recommended for the primary and secondary prevention of bleeding [2, 3]. The beta1-blockers and isosorbide mononitrate can reduce portal pressure [4]. Statins are associated with a lower risk of hepatic decompensation and mortality in patients with chronic liver diseases and liver cirrhosis [5, 6]. On the other hand, antiplatelet therapy can increase the risk of bleeding in patients with vitamin K-dependent coagulopathies, thrombocytopenia, esophageal varices, and gastric ulcers. Ranolazine is contraindicated in patients with liver cirrhosis, due to the increase of C_max_ (maximum serum concentration) by 80% in cirrhotic patients with moderate hepatic impairment. Furthermore, the risk of long QT interval rises threefold. Rivaroxaban should not be used in patients with Child–Pugh class B liver cirrhosis because of increase the drug exposure more than twice [7, 8].

**Table 3:** FORCOM drug classification for treatment of coronary artery disease in patients with liver cirrhosis

Class	Drugs
A	Nonselective beta-blockers (nadolol, propranolol, carvedilol)
B	Beta_1_-blockers, isosorbide mononitrate, statins
C	Calcium antagonists, molsidomine, nitrates, nicorandil, trimetazidine
D	Antiplatelets
X	Ranolazine, rivaroxaban

The FORCOM classification of psychopharmacological drugs in patients with coronary artery disease is shown in Table 4. Antidepressants and anxiolytics are used in patients with coronary artery disease to treat depressive and anxiety disorders reducing the quality of life to a greater extent than the somatic diseases. The medications with the lowest cholinolytic, arrhythmogenic, and hemodynamic adverse effects are usually selected for patients with coronary artery disease. The safety of selective serotonin reuptake inhibitors (sertraline, citalopram, and escitalopram) and mirtazapine were confirmed in patients after myocardial infarction by reliable studies [9–11]. Sertraline which is the most studied in randomized trails drug have a weak effect on QT interval than citalopram and escitalopram, and fewer interactions with proton pump inhibitors than escitalopram [12, 13]. Treatment of depression with escitalopram reduced the risk of cardiac events in the randomized EsDEPACS trial including 300 patients after acute coronary syndrome [14].

**Table 4:** FORCOM drug classification for treatment of mental disorders in patients with coronary artery disease

Class	Drugs
C	Benzodiazepines, selective serotonin reuptake inhibitors, mirtazapine, trazodone
D	Antipsychotics, monoamine oxidase inhibitors, serotonin-norepinephrine reuptake inhibitors, mianserin, trazodone
X	Tricyclic antidepressants

It must be taken into account that selective serotonin reuptake inhibitors increase the risk of bleeding in patients taking these drugs combined with aspirin or dual antiplatelet therapy [15, 16]. Venlafaxine is associated with some undesirable cardiovascular effects, including hypertension and orthostatic hypotension [17]. The effect of monoamine oxidase inhibitors, mianserin, and trazodone in patients with coronary artery diseases has not been established. Tricyclic antidepressants are contraindicated in patients with heart diseases due to cardiac adverse effects, including sinus tachycardia, postural hypotension, and electrophysiological changes (prolongation of QRS, PR, and QT intervals) even at therapeutic dosages. There are conflicting estimates of the relationship between antipsychotics and the risk of myocardial infarction [18, 19]. It should be taken into account that antipsychotics may increase the risk of ventricular tachyarrhythmias and sudden cardiac arrest [20, 21].

In this article, we are the first to highlight the common principles of the drug classification system. Due to the lack of reliable studies, the attribution of drugs to one class or another may be debated and improved.

Therefore, the FORCOM classification system may be useful to make consistent the positive and negative effects of drugs on comorbidities, to unify the existing narrow classifications, to compare different expert opinions, to simplify available information in clinical guidelines, and to help practitioners in the selection of efficacious and safe drugs for patients with multiple comorbidities.
